# Measures of Effectiveness, Efficiency, and Quality of Telemedicine in the Management of Alcohol Abuse, Addiction, and Rehabilitation: Systematic Review

**DOI:** 10.2196/13252

**Published:** 2020-01-31

**Authors:** Clemens Scott Kruse, Kimberly Lee, Jeress B Watson, Lorraine G Lobo, Ashton G Stoppelmoor, Sabrina E Oyibo

**Affiliations:** 1 School of Health Administration Texas State University San Marcos, TX United States

**Keywords:** telemedicine, telehealth, mHealth, alcohol abuse, rehabilitation, alcohol use disorder

## Abstract

**Background:**

More than 18 million Americans are currently suffering from alcohol use disorder (AUD): a compulsive behavior of alcohol use as a result of a chronic, relapsing brain disease. With alcohol-related injuries being one of the leading causes of preventable deaths, there is a dire need to find ways to assist those suffering from alcohol dependence. There still exists a gap in knowledge as to the potential of telemedicine in improving health outcomes for those patients suffering from AUD.

**Objective:**

The purpose of this systematic review was to evaluate the measures of effectiveness, efficiency, and quality that result from the utilization of telemedicine in the management of alcohol abuse, addiction, and rehabilitation.

**Methods:**

This review was conducted utilizing the Preferred Reporting Items for Systematic Reviews and Meta-Analyses guidelines. The articles used in this analysis were gathered using keywords inclusive of both *telemedicine* and *alcohol abuse*, which were then searched in the Cumulative Index to Nursing and Allied Health Literature, Cochrane, and MEDLINE (PubMed) databases. A total of 22 articles were chosen for analysis.

**Results:**

The results indicated that telemedicine reduced alcohol consumption. Other common outcomes included reduced depression (4/35, 11%), increased patient satisfaction (3/35, 9%), increase in accessibility (3/35, 9%), increased quality of life (2/35, 6%), and decreased cost (1/35, 3%). Interventions included mobile health (11/22, 50%), electronic health (6/22, 27%), telephone (3/33, 14%), and 2-way video (2/22, 9%). Studies were conducted in 3 regions: the United States (13/22, 59%), the European Union (8/22, 36%), and Australia (1/22, 5%).

**Conclusions:**

Telemedicine was found to be an effective tool in reducing alcohol consumption and increasing patients’ accessibility to health care services or health providers. The group of articles for analysis suggested that telemedicine may be effective in reducing health care costs and improving the patient’s quality of life. Although telemedicine shows promise as an effective way to manage alcohol-related disorders, it should be further investigated before implementation.

## Introduction

### Background

More than 18 million Americans are currently suffering from alcohol use disorder (AUD) [1]. The National Institute on Alcohol Abuse and Alcoholism defines AUD as a *chronic relapsing brain disease in which a person or individual displays compulsive alcohol use, loss of control in regards to alcohol intake, and a negative emotional state when not using* [2]. An individual’s consistent engagement in the use of alcohol may prove harmful not only to the individual’s health but to the health of others as well.

A growing concern in the health care field is the alarming number of preventable deaths that result from alcohol-related injuries. It is estimated that excessive alcohol use is responsible for approximately 88,000 deaths and approximately 2.5 million potential life years lost annually in the United States and in the European Union; approximately 1 in 4 deaths of males aged 15 to 39 years are because of alcohol [3,4]. Owing to the substantial health and economic barriers that result from alcohol dependence, there exists a dire need for an alternative and innovative solution for managing alcohol abuse, addiction, and rehabilitation.

Telemedicine, specifically videoconferencing, is identified as a successful tool in reducing the effects of adults suffering from AUD, but videoconferencing is only one small aspect of telemedicine that can offer assistance to this condition [5]. Telemedicine, as defined by the World Health Organization is:

The delivery of health care services, where distance is a critical factor, by all health care professionals using information and communication technologies for the exchange of valid information for diagnosis, treatment and prevention of disease and injuries, research and evaluation, and for the continuing education of health care providers, all in the interests of advancing the health of individuals and their communities [6].

The use of telemedicine in treating alcohol dependence helps increase the patient’s access to providers as well as increasing social support outside of the health care setting [5]. Both factors have demonstrated effectiveness in managing addiction and/or preventing relapse. Telemedicine has the ability to reduce obstacles such as geographical locations and time while still continuing to deliver the same (or better) quality health care [5]. Despite these findings, there still is a gap in knowledge as to the potential of telemedicine-based treatments in improving health outcomes in AUD patients.

A review was conducted on this topic in 2012 [7]. It provided an extensive review of literature (n=50); studies were conducted in 7 countries; interventions reported were telephone and voice response, videoconferencing, text messaging, Web, email, and chat; and outcomes reported were participation, substance use, satisfaction, and resource utilization. However, technology has evolved a great deal in telecommunications since 2012, and some researchers suggest reviews should be repeated after 2 years [8]. Another review was conducted more recently in Australia [9]. It only analyzed 19 articles and focused purely on mobile apps as an intervention in 1 country [9].

### Objective

The purpose of this systematic review was to analyze and evaluate current literature in regard to the measures of effectiveness, efficiency, and quality that result from the use of telemedicine in managing alcohol abuse, addiction, and rehabilitation in patients. Effectiveness was measured through outcomes. Efficiency was measured through cost. Quality was measured in terms of safety, timeliness, access, patient satisfaction, or quality of life.

## Methods

### Protocol and Registration

The research process was structured following the Kruse Protocol for Writing Systematic Reviews [10] and the Assessment for Multiple Systematic Reviews (AMSTAR) [11]. Findings were reported in accordance with the Preferred Reporting Items for Systematic Reviews and Meta-Analyses (PRISMA) [12]. The PRISMA checklist for this review is listed in Multimedia Appendix 1. The authors started the process with the identification of a specific set of key terms intended to produce a list of inclusive articles from a variety of domains that were directly related to the topic of discussion. Both *telemedicine* and *alcohol abuse* were searched on MEDLINE (PubMed) using the US National Library of Medicine’s Medical Subject Headings (MeSH). A total of 5 subheadings were identified under the search of *telemedicine*, and 13 subheadings were identified for *alcohol abuse*. For the purposes of this systematic review, these 18 subheadings were selected as the final set of key terms to be used for searches within the research databases. To ensure we captured at least 10 years of research, searches were conducted on December 31, 2018, and the time period of inclusion was December 1, 2009 to December 31, 2018.

The subheadings were separated utilizing Boolean operators to ensure the search produced articles that are inclusive of both alcohol abuse and telemedicine or terms related to those topics. See Multimedia Appendix 2 for the exact Boolean phrase used in all databases.

### Eligibility Criteria, Information Sources, and Search

A total of 3 research databases were chosen: Cumulative Index to Nursing and Allied Health Literature (CINAHL), PubMed (MEDLINE), and Cochrane. These databases were chosen because of their wide availability, following the Kruse Protocol for writing a systematic review, and because they are recommended by the National Institutes of Health [13].

Articles were eligible for analysis if they were published in the last 10 years and included information on the use of telemedicine for the treatment of AUD. We preferred the articles also included data on outcomes, but that was not an eligibility criterion.

**Figure 1:**
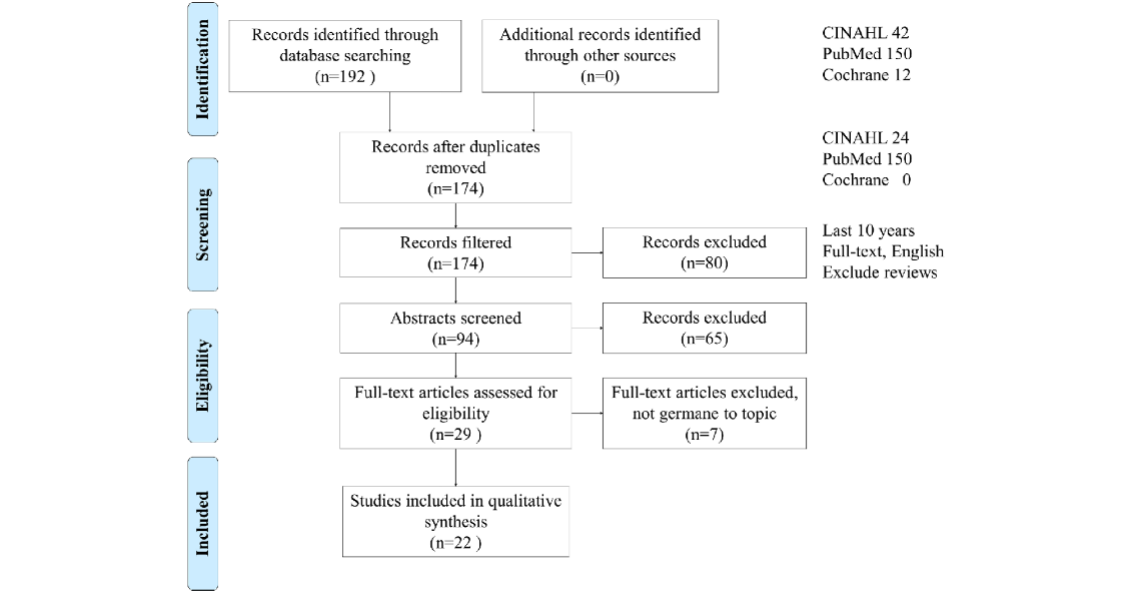
Preferred Reporting Items for Systematic Reviews and Meta-Analyses flow diagram. CINAHL: Cumulative Index of Nursing and Allied Health Literature.

Using the key terms from MeSH, the initial search in the CINAHL yielded a total of 42 articles, the PubMed database yielded 150 articles, and Cochrane yielded 12. The search process and criteria are visually represented in Figure 1. To limit the search to recent and relevant articles, filters were applied to both CINAHL, PubMed, and Cochrane database searches. In CINAHL, MEDLINE was excluded to help eliminate duplicates. Filters were applied to both meet our acceptance criteria of the last 10 years and to ensure articles were available (full text, English, no reviews). A total of 94 articles remained for the next step in the process.

### Study Selection and Data Collection Process

The next step of the systematic review process consisted of a screening of abstracts by the authors. The 94 abstracts were divided between 5 authors to be carefully screened according to the objective statement. Each abstract was screened by at least two authors, and a final consensus meeting was held to finalize the selection of articles to be included in the systematic review. During this process, a total of 65 articles were excluded for not being germane, leaving a total of 29 articles.

The 29 articles were read in their entirety by at least two reviewers and excluded if articles were not related to the objective statement, were study protocols, or were systematic reviews. Similar to the screening process, articles were divided among the 6 reviewers for analysis commensurate with our objective statement. Workload was divided among 6 authors following the Kruse Protocol [10]: JW, LL, AS, and SO each analyzed 10 articles, KL analyzed 5 articles, and CSK analyzed all 29 articles. This process left a total of 22 articles for inclusion in the group for analysis. Figure 1 illustrates the screening and selection process.

### Level of Agreement

A kappa statistic was calculated to assess the level of agreement between the authors. The calculated kappa score was 0.85 indicating strong agreement on which articles should be included in the group for analysis [14-16]. The closer this score is to 1, the stronger the agreement.

### Data Items, Summary Measures, Synthesis of Results, Bias, and Additional Analysis

During the analysis phase, data items were extracted such as participants, intervention, comparison method, outcomes, as well as measures of effectiveness, efficiency, and quality. Each article was analyzed by at least two reviewers, and independent observations were made. A consensus meeting was held to discuss observations and distill observations to themes. On the basis of the combined set of observations and identified themes, a second read of articles was conducted and another consensus meeting held to ensure an exhaustive data collection. Themes were analyzed for trends. Affinity matrices were created to describe frequencies of data. The frequency of occurrence was recorded for all articles. Multiple articles mentioned multiple themes. Observations of potential bias were recorded.

## Results

### Study Selection, Study Characteristics, Results of Studies, and Synthesis of Results

Multimedia Appendix 3 lists a detailed summary of the analysis of the 22 articles in the review.

All articles in the analysis discuss telemedicine for the treatment of AUD [17-38].

### Additional Analysis

After performing a punctilious data extraction process, the authors were able to identify several potential outcomes that were used to measure the effectiveness of telemedicine in the management of alcohol use, addiction, and rehabilitation: reduction in alcohol consumption, increase in patient satisfaction, reduction in symptoms of depression, increased accessibility, and decreased cost. The authors selected a total of 7 outcomes as evaluation criteria for article analysis in a total of 35 occurrences (number of times the theme was addressed). Articles can include multiple themes. An affinity matrix of these themes is listed in Table 1.

Out of the outcomes identified, a total of 16 out of 35 (46%) occurrences mentioned that there was a statistically significant reduction in the participants’ alcohol consumption [17,18,20-30,32,35,38]. A total of 6 of 35 (17%) showed an increase in cognition [19,20,31,33,36]. A total of 4 of 35 (11%) showed a decrease in depressive symptoms [17,21,26,27]; and 2 themes were each mentioned 3 of 35 (9%) occurrences: increased patient satisfaction [18,22,25] and increased accessibility [17,20,24]. The latter included easily accessible tools for the participants to use that were not associated with the negative stigma of being treated for alcohol abuse. In all, 2 of 35 (6%) occurrences were increased quality of life [20,26], and 1 of 35 (3%) was cost-effectiveness of the intervention [37].

A total of 4 technological interventions were identified (summarized in Table 2). The intervention most often identified in the literature was a mobile app or SMS, which are text messages. This intervention was identified in 11 of the 22 articles (50%) [18,19,23-25,29,31,36]. The next most identified intervention was Web-based (electronic health, eHealth), which occurred in 6 of 22 articles (27%) [17,20,26,37]. One intervention occurred in 3 of the 22 articles (14%): phone-based (voice) [22,35,38]. Finally, 2-way video occurred in 2 of 22 articles (9%) [21,30].

Researchers from 6 different countries generated articles (summarized in Table 3). Researchers in the United States generated more than half the articles, producing 13 of 22 articles (59%) [19-23,28-32,35,38]. Researchers from European countries produced 8 articles including Sweden [24,26], Portugal [33,34], the United Kingdom [18], Spain [25], Germany [17], and the Netherlands [37]. Research from Australia occurred once in the literature [27].

Telemedicine showed positive medical outcomes (effectiveness) in 77% (17/22) of the articles analyzed and resulted in no statistical significance in improvement in the remaining 23% (5/22). There were no observations where telemedicine resulted in a decrease in medical outcome or effectiveness.

**Table 1:** Themes observed in the literature (N=35).

Themes	Studies (references)	Frequency, n (%)	
Reduced alcohol consumption	[17,18,20-30,32,35,38]	16 (46)	
Increased cognition	[19,20,31,33,36]	6 (17)	
Reduced depression	[17,21,26,27]	4 (11)	
Increased patient satisfaction	[18,22,25]	3 (9)	
Increased accessibility	[17,20,24]	3 (9)	
Increased quality of life	[20,26]	2 (6)	
Decreased cost	[37]	1 (3)	

**Table 2:** Interventions observed in the literature (N=22).

Intervention	Studies (references)	Frequency, n (%)	
Mobile health	[18,19,23-25,29,31,36]	11 (50)	
Electronic health	[17,20,26,37]	6 (27)	
Telephone	[22,35,38]	3 (14)	
2-way video	[21,30]	2 (9)	

**Table 3:** Summary of country of origin of research (N=22).

Country	Studies (references)	Frequency, n (%)	
The United States	[19-23,28-32,35,38]	13 (59)	
European Union	[17,18,24-26,33,37]	8 (36)	
Australia	[27]	1 (5)	

## Discussion

### Summary of Evidence

The key intent in this systematic review was to identify measures of effectiveness, efficiency, and quality of telemedicine in managing conditions of AUD or some sort of substance dependence. A total of 7 measures of effectiveness and 4 categories of technological intervention from 8 countries were identified in 22 articles. The intervention of technology was associated with positive medical outcomes in 17 of the 22 articles [17-21,23-27,29-33,35,36]. In the other 5 articles, it equaled the outcome of the control group [22,28,34,37,38]. Most importantly, technological interventions enabled patients to meet their goals with a tool that could be used independent of the health care facility, without the negative stigma associated with alcohol and drug abuse or addition and depressive or posttraumatic stress disorder symptoms.

Subjects, including patients, clients, students, or employees, reported high levels of satisfaction with the technological interventions for several reasons. The technology solutions enabled a variety of positive aspects of care: self-management of subjects’ symptoms or conditions, the technology solutions were self-paced, the technology solutions were both synchronous and asynchronous with providers, and technology solutions were based on subjects’ preferences. Individuals did not have to travel to the provider or counselor, reducing costs, and addressing concerns over a stigma of being treated for AUD [26]. Subjects accessed solutions via mobile health and eHealth around the clock, 24×7, rather than spending valuable time making and traveling to and from onsite appointments. The telehealth solutions enabled real-time management of temptations and other problems through interactive and often customizable feedback to help patients change their behavior.

Beyond the scope of this study and yet an important consideration, providers incurred costs for the technological interventions reviewed in this study, including initial development, capital securement, supportive programming, administration, personnel, and time and resources for ongoing delivery and aftercare. However, organizations’ ability to treat and provide valuable resources to recipients of care outside the brick and mortar far exceeded the cost of the intervention. Organizations were able to expand practice without expanding square footage, and eHealth solutions continued to provide care outside the boundaries of 8 am to 5 pm, a traditional treatment day. An organization with the treatment of alcohol and drug abuse or addiction and depressive symptoms should strongly consider a technological intervention because it would enable the organization to meet this strategic goal without another product line, additional provider staff, or clinic space.

The implementation of telemedicine as an alcohol management treatment option has demonstrated great promise in reducing alcohol consumption and positively affecting other factors associated with alcohol use and improved patient outcomes. Alcohol management is the treatment of alcohol abuse through moderation. It addresses the highly complex and personal issue that alcoholism can create [39]. Alcohol management helps an individual drink in moderation when abstinence programs are ineffective.

The 22 articles analyzed included participants from several countries. The authors identified a total of 7 characteristics for use in measuring telemedicine’s effectiveness in AUD patients. Most of the articles mentioned a decrease in alcohol consumption (16/22) and an increase in accessibility (3/22). A social stigma was mentioned that is a cause for concern, and it supports the adoption of telemedicine [26]. The fear is being seen in or around the clinic that treats AUD. Treatment through telemedicine overcomes this stigma by enabling patients to be treated virtually. Other barriers were listed within the articles but were not listed enough times to be able to state that a correlation exists between those barriers and the adoption of telemedicine in treating AUDs.

It is unfortunate that so few articles qualified for analysis based on our selection criteria. To do this study over again, we would expand our selection criteria to enable a larger number of articles to be included in the analysis. The larger number of articles would strengthen the results and associated conclusions and give us a stronger indication of external validity.

Of interesting note, the countries publishing articles on AUD are developed countries. It is unlikely that developing countries do not experience difficulties with AUD. Instead, it is more likely that these countries are not devoting resources to research the topic.

### Strategic Leadership and Cultural Implications

Our systematic review illuminated the need for strategic leadership in planning care environments to include innovative, effective, and efficient solutions such as telemedicine. Leaders and experts in the field of health care emphasize the necessity of delivering value-based care according to trends as identified by over 2000 health care leaders in a 2017 survey [40]. Chief executives of hospitals and other care organizations anticipate that consumers will increasingly expect autonomous, user-friendly, and empathetic care, and consumers’ expectations will coincide with the necessity of organizations to deliver value, equity, safety, transformation, affordability, innovation, and efficiency [40]. A consistency of subjects experiencing enhanced autonomy and satisfaction occurred in this systematic review, supporting predicted trends in consumers’ expectations [17,18,20-22,25-27].

One of the increasingly frequent realities, the need for cybersecurity, will create a culture of increased trust with consumers and will also serve to address one of the cited barriers to traditional care in this study, threat of social exposure and lack of confidentiality around participation in electronic telehealth around a socially sensitive condition, alcohol misuse, abuse, and hazardous lifestyles [26,40]. Increased leadership strategy around creating discipline in behaviors, practices, and process improvement with the intent to protect private data will serve to strengthen the organizational culture and hygiene.

Strategic leaders considering future trends also set a vision of redesigned, decentralized, nontraditional, and remote health care facilities to better meet the needs of consumers, better protect patients and staff, and better prepare for sustainability with greater unpredictability of reimbursement and regulations [40].

Organizational leaders also have the responsibility to incorporate telehealth interventions to not only better reach individuals afflicted by AUDs but also to reduce the societal health burden. To this point, the World Health Organization in its Sustainable Development Goal Agenda of 2030 cites the harmful use of alcohol as a leading risk factor for worldwide health and includes expectations of organizations to assess and implement cost-efficient and multimedia approaches to impact the global issue [41].

### Limitations of the Study

The limited number of articles selected in the group for analysis provided was a limitation. With smaller samples, it is possible that the results found within each study cannot be broadly applied to the population. In addition, self-reporting was heavily relied on within these studies and ultimately could have led to self-report bias when patients reported back to the researchers.

### Strengths of Our Review

A strength to be considered is the process used to select the final 22 articles for the review. Utilizing the US National Library of Medicine’s MeSH enabled an exhaustive search of the literature. Adopting the technique from AMSTAR to use multiple, independent reviewers for both screening abstracts and analyzing articles reduces selection bias [11]. Furthermore, a composite kappa score of 0.85 reflected strong agreement among the authors on their choice of articles based on the objective statement [16]. The kappa score is a measure of agreement between observers [14]. The score establishes confidence in the articles chosen and their relation to the objective statement as there were no articles included in the review that were not agreed upon by 2 or more of the authors. There was also agreement among the authors of the articles chosen that further research is needed to identify those interventions with the strongest level of effectiveness.

### Conclusions

Overall, there are many potential applications for telemedicine in improving patient outcomes and strategy in the delivery of care across diverse environments. Telemedicine has the ability to expand the scope of health care by allowing patients the ability to connect to health care providers without the barriers of proximity, without the negative stigma associated with alcohol or drug addiction or mental health, and without the expense of driving to the clinic for an in-person visit.

Telemedicine can allow patients greater access to their provider in times of need. One of the key necessities for successful alcoholism recovery is behavioral therapy and counseling for the patient [39]. The concept used is that the increased communication between the patient and provider will aid the patient in overcoming the barriers associated with alcohol recovery and in turn reduce the number of alcohol-related deaths. With further investigation and research, the use of telemedicine in managing AUDs may be strategically achievable soon.

## Appendix

Multimedia Appendix 1 Preferred Reporting Items for Systematic Reviews and Meta-Analyses checklist.

Multimedia Appendix 2 Boolean search string.

Multimedia Appendix 3 Summary of evidence.

## Abbreviations

AMSTAR: Assessment for Multiple Systematic Reviews
AUD: alcohol use disorder
CINAHL: Cumulative Index to Nursing and Allied Health Literature
eHealth: electronic health
MeSH: Medical Subject Headings
PRISMA: Preferred Reporting Items for Systematic Reviews and Meta-Analyses

## References

[1] MedlinePlus.

[2] National Institute on Alcohol Abuse and Alcoholism (NIAAA).

[3] Centers for Disease Control and Prevention.

[4] World Health Organization.

[5] Frueh BC, Henderson S, Myrick H (2005). Telehealth service delivery for persons with alcoholism. J Telemed Telecare.

[6] World Health Organization (2010). Telemedicine: Opportunities and Developments in Member States.

[7] Young LB (2012). Telemedicine interventions for substance-use disorder: a literature review. J Telemed Telecare.

[8] Garner P, Hopewell S, Chandler J, MacLehose H, Schünemann HJ, Akl EA, Beyene J, Chang S, Churchill R, Dearness K, Guyatt G, Lefebvre C, Liles B, Marshall R, García LM, Mavergames C, Nasser M, Qaseem A, Sampson M, Soares-Weiser K, Takwoingi Y, Thabane L, Trivella M, Tugwell P, Welsh E, Wilson EC, Schünemann HJ, Panel for updating guidance for systematic reviews (PUGs) (2016). When and how to update systematic reviews: consensus and checklist. Br Med J.

[9] Choo CC, Burton AA (2018). Mobile phone apps for behavioral interventions for at-risk drinkers in Australia: literature review. JMIR Mhealth Uhealth.

[10] Kruse CS (2019). Writing a systematic review for publication in a health-related degree program. JMIR Res Protoc.

[11] Shea BJ, Grimshaw JM, Wells GA, Boers M, Andersson N, Hamel C, Porter AC, Tugwell P, Moher D, Bouter LM (2007). Development of AMSTAR: a measurement tool to assess the methodological quality of systematic reviews. BMC Med Res Methodol.

[12] Moher D, Liberati A, Tetzlaff J, Altman DG, PRISMA Group (2009). Preferred reporting items for systematic reviews and meta-analyses: the PRISMA statement. PLoS Med.

[13] National Institutes of Health.

[14] Cohen J (1960). A coefficient of agreement for nominal scales. Educ Psychol Meas.

[15] Light RJ (1971). Measures of response agreement for qualitative data: some generalizations and alternatives. Psychol Bull.

[16] McHugh ML (2012). Interrater reliability: the kappa statistic. Biochem Med (Zagreb).

[17] Boß L, Lehr D, Schaub MP, Castro RP, Riper H, Berking M, Ebert DD (2018). Efficacy of a web-based intervention with and without guidance for employees with risky drinking: results of a three-arm randomized controlled trial. Addiction.

[18] Attwood S, Parke H, Larsen J, Morton KL (2017). Using a mobile health application to reduce alcohol consumption: a mixed-methods evaluation of the drinkaware track & calculate units application. BMC Public Health.

[19] Glass JE, McKay JR, Gustafson DH, Kornfield R, Rathouz PJ, McTavish FM, Atwood AK, Isham A, Quanbeck A, Shah D (2017). Treatment seeking as a mechanism of change in a randomized controlled trial of a mobile health intervention to support recovery from alcohol use disorders. J Subst Abuse Treat.

[20] Acosta MC, Possemato K, Maisto SA, Marsch LA, Barrie K, Lantinga L, Fong C, Xie H, Grabinski M, Rosenblum A (2017). Web-delivered CBT reduces heavy drinking in OEF-OIF veterans in primary care with symptomatic substance use and PTSD. Behav Ther.

[21] Jaconis M, Santa Ana EJ, Killeen TK, Badour CL, Back SE (2017). Concurrent treatment of PTSD and alcohol use disorder via telehealth in a female Iraq veteran. Am J Addict.

[22] Rose GL, Badger GJ, Skelly JM, MacLean CD, Ferraro TA, Helzer JE (2017). A randomized controlled trial of brief intervention by interactive voice response. Alcohol Alcohol.

[23] Muench F, van Stolk-Cooke K, Kuerbis A, Stadler G, Baumel A, Shao S, McKay JR, Morgenstern J (2017). A randomized controlled pilot trial of different mobile messaging interventions for problem drinking compared to weekly drink tracking. PLoS One.

[24] Gajecki M, Andersson C, Rosendahl I, Sinadinovic K, Fredriksson M, Berman AH (2017). Skills training via smartphone app for university students with excessive alcohol consumption: A randomized controlled trial. Int J Behav Med.

[25] Barrio P, Ortega L, López H, Gual A (2017). Self-management and shared decision-making in alcohol dependence via a mobile app: a pilot study. Int J Behav Med.

[26] Johansson M, Sinadinovic K, Hammarberg A, Sundström C, Hermansson U, Andreasson S, Berman AH (2017). Web-based self-help for problematic alcohol use: a large naturalistic study. Int J Behav Med.

[27] Deady M, Mills KL, Teesson M, Kay-Lambkin F (2016). An online intervention for co-occurring depression and problematic alcohol use in young people: Primary outcomes from a randomized controlled trial. J Med Internet Res.

[28] Campbell AN, Nunes EV, Matthews AG, Stitzer M, Miele GM, Polsky D, Turrigiano E, Walters S, McClure EA, Kyle TL, Wahle A, van Veldhuisen P, Goldman B, Babcock D, Stabile PQ, Winhusen T, Ghitza UE (2014). Internet-delivered treatment for substance abuse: a multisite randomized controlled trial. Am J Psychiatry.

[29] Stoner SA, Arenella PB, Hendershot CS (2015). Randomized controlled trial of a mobile phone intervention for improving adherence to naltrexone for alcohol use disorders. PLoS One.

[30] Staton-Tindall M, Havens JR, Webster JM, Leukefeld C (2014). METelemedicine: a pilot study with rural alcohol users on community supervision. J Rural Health.

[31] Chih M (2014). Exploring the use patterns of a mobile health application for alcohol addiction before the initial lapse after detoxification. AMIA Annu Symp Proc.

[32] Chih M, Patton T, McTavish FM, Isham AJ, Judkins-Fisher CL, Atwood AK, Gustafson DH (2014). Predictive modeling of addiction lapses in a mobile health application. J Subst Abuse Treat.

[33] Gamito P, Oliveira J, Lopes P, Brito R, Morais D, Caçoete C, Rebelo S, Silva D, Deus A (2016). Cognitive stimulation through mHealth-based program for patients with alcohol dependence syndrome: a randomized controlled study. J Pain Manag.

[34] Gamito P, Oliveira J, Lopes P, Brito R, Morais D, Silva D, Silva A, Rebelo S, Bastos M, Deus A (2014). Executive functioning in alcoholics following an mHealth cognitive stimulation program: randomized controlled trial. J Med Internet Res.

[35] Rose GL, Skelly JM, Badger GJ, Naylor MR, Helzer JE (2012). Interactive voice response for relapse prevention following cognitive-behavioral therapy for alcohol use disorders: a pilot study. Psychol Serv.

[36] McTavish FM, Chih M, Shah D, Gustafson DH (2012). How patients recovering from alcoholism use a smartphone intervention. J Dual Diagn.

[37] Smit F, Lokkerbol J, Riper H, Majo MC, Boon B, Blankers M (2011). Modeling the cost-effectiveness of health care systems for alcohol use disorders: how implementation of eHealth interventions improves cost-effectiveness. J Med Internet Res.

[38] McKay JR, van Horn D, Oslin DW, Ivey M, Drapkin ML, Coviello DM, Yu Q, Lynch KG (2011). Extended telephone-based continuing care for alcohol dependence: 24-month outcomes and subgroup analyses. Addiction.

[39] Alcohol | American Addiction Centers.

[40] Society for Healthcare Strategy and Market Development.

[41] World Health Organization (2018). Global Status Report on Alcohol and Health 2018.

