# Supplementary material for: Measures of Effectiveness, Efficiency, and Quality of Telemedicine in the Management of Alcohol Abuse, Addiction, and Rehabilitation: Systematic Review
Source: J Med Internet Res. 2020 Jan 31;22(1):e13252. doi: 10.2196/13252 (PMC7055825; doi:10.2196/13252)
Supplement: Multimedia Appendix 2 [file jmir_v22i1e13252_app2.docx]

# Appendix 2

**Boolean phrase used in search**

(“Alcohol Dependence” OR “Dependence, Alcohol” OR “alcohol Addiction” OR “Addiction, Alcohol” OR “Alcoholic Intoxication, Chronic” OR “Chronic Alcoholic Intoxication” OR “Intoxication, Chronic Alcoholic” OR “Alcohol Use Disorder” OR “Alcohol Use Disorders” OR “Use Disorder, Alcohol” OR “Use Disorders, Alcohol” OR “ Alcohol Abuse” OR “Abuse, Alcohol”) AND (“Mobile Health” OR “ Abuse, Alcohol” ) AND (“Mobile Health” OR “Health, Mobile” OR mHealth OR Telehealth OR eHealth)
