# Supplementary material for: Measures of Effectiveness, Efficiency, and Quality of Telemedicine in the Management of Alcohol Abuse, Addiction, and Rehabilitation: Systematic Review
Source: J Med Internet Res. 2020 Jan 31;22(1):e13252. doi: 10.2196/13252 (PMC7055825; doi:10.2196/13252)
Supplement: Multimedia Appendix 3 [file jmir_v22i1e13252_app3.docx]

Table 1. Articles and main findings.

| Authors | Participants | Intervention | Statistical process | Medical outcome | Themes of effectiveness, efficiency, or quality | Specifics of intervention | Country | Study design |
| --- | --- | --- | --- | --- | --- | --- | --- | --- |
| Boß et al [17] | n=434; adults (178 male 41%, 256 female 59%); mean age 47 years (gender and attrition not provided) | Electronic health (eHealth) | - Markov Chain Monte Carlo multivariate imputation algorithm - Pearson chi-square - All groups showed reductions of mean weekly serum uric acid (SUA) after 6 weeks (unguided: −8.0, guided: −8.5, control: −3.2). There was no significant difference between the unguided and guided intervention (*P*=.324). | Significantly reduced consumption of standard units of alcohol and improved mental health and work-related outcomes. The intervention groups showed significant reductions in SUA consumption after 6 months (*B*=−5.72; 95% CI −7.71 to −3.73; *P*<.001). | - Reduced alcohol consumption - Increased accessibility - Reduced depression | - Normative feedback - Motivational interviewing - Emotional regulation | Germany | Randomized controlled trial (RCT), 3 arm, I1=146, I2=144, C=144; small effect size |
| Attwood et al [15] | n=100,262; adults (43,061 male 42.9%, 57,201 female 57.1%); gender not provided; high attrition after 1 week | Mobile heath (mHealth) | - Descriptives - Analysis of variance (ANOVA; *P*<.001) | Significant reduction in alcohol consumption; the number of “no drink days” per week increased (on-boarding “typical week”=2.7 days per week; week 1=3.3 days per week; week 4=3.1 days per week; week 12=3.1 days per week). | - Reduced alcohol consumption - Increased patient satisfaction | - Self-monitoring - Customized SMS | The United Kingdom | Mixed-method, no effect size reported |
| Glass et al [19] | n=349; adults (212 male 60.7%, 137 female 29.3%); mean age 38.3 years; 293 white 84.0%, 45 black 12.9%; attrition not provided | mHealth | Log-linear mixed effects and logistic model for abstinence (odds ratio, OR=2.14, 95% CI 1.27 to 3.61) | Significantly reduced risky drinking days by 11% over the control arm. | Increased cognition | Addition Comprehensive Health Enhancement Support System (A-CHESS) followed by questionnaires and feedback | The United States | RCT, equal arms; small effect size |
| Acosta et al [20] | n=162; adults (151 male 93.2%, 11 female 6.8%); mean age 34 years; 138 white 85.2%; attrition not provided | eHealth | - Pearson chi-square - *P*<.05 | Significant results for heavy drinking but not Post-Traumatic Stress Disorder (PTSD) or quality of life. 36.9%>drinking, 23.0%=drinking, and 39.4%<drinking. 5.1%>drug use, 83.1%=use, and 21.9%<use. | - Reduced alcohol consumption - Increased cognition - Increased accessibility - Increased quality of life (QOL) | Thinking forward (Web-based cognitive behavioral therapy, CBT) | The United States | RCT, equal arms; no effect size reported |
| Jaconis et al [21] | n=1; black female | 2-way video | - Descriptives - Mean comparisons | Significant reduction in alcohol consumption as well as PTSD and depressive symptoms. 63% reduction in symptoms endorsed on the Post-Traumatic Stress Disorder Checklist- Military and an 82% reduction on the Clinician-Administered PTSD Scale. The patient’s score of 48 at baseline on the Beck Depression Inventory indicated “severe” symptoms, and at 6-month follow-up, her score was 6. Drinking reduced from 6 per day down to 1 per day. | - Reduced alcohol consumption - Reduced depression | 2-way video and CPOE | The United States | Case study; no effect size reported |
| Rose et al [22] | n=1705; adults (818 male 48.0%, 887 female 52.0%); mostly Caucasian; attrition same between groups (control 232, intervention 260) | Telephone | - ANOVA - Paired *t* test (*P*<.001) | Significant reduction in drinking for both groups (no statistically significant difference), and for those with alcohol use disorder, the intervention group showed significant decrease in drinking outcomes at the 3-month assessment. | - Reduced alcohol consumption - Increased patient satisfaction | 12 weekly 90-min group CBT sessions, interactive voice response-brief intervention | The United States | RCT, 2 arms, I=938, C=917; small effect size |
| Muench et al [23] | n=145 (36 male 25.1%, 109 female 74.9%). Mean age 43.2 years, 136 white, 3 Hispanic, 3 others, 2 black, 1 Asian, 7 attrited. | mHealth | - Poisson regression models. - The partial correlation between subjective overall change and actual change in SDD was significant, r=0.49; *P*<.0001. | Significant reduction in alcohol consumption (number of drinks and heavy drinking days) for all arms except Gain Frame group, in comparison with the control. The TA group showed the largest effects, *b*=−0.40; *P*<.001. | Reduced alcohol consumption | Tailored SMS, and adaptive SMS | The United States | Single-blind, RCT pilot study, 5 arms, I1=31, I2=26, I3=30, I4=31, C=28; medium effect size |
| Gajecki et al [24] | n=330; adult students (102 male 30.9%, 228 female 69.1%); mean age 25.4 years; race not disclosed; attrition arm 1, 2 and control=22, 26, and 4, respectively | mHealth | - Cronbach alpha (α=.83) and Z-scores. Secondary analyses showed reductions for the intervention group in quantity of drinking at first follow-up (−4.76, 95% CI −6.67 to −2.85, *Z*=−2.09; *P*=.037) - And in frequency of drinking at both follow-ups (−0.83, 95% CI −1.14 to −0.52, *Z*=−2.04, *P*=.041; −0.89, 95% CI −1.16 to −0.62, *Z*=−2.12, *P*=.034). | Significant reduction in number of drinks and frequency of drinking. The OR for not having excessive alcohol consumption among men in the intervention group compared with male controls was 2.68, (95% CI 1.37 to 5.25; *Z*=2.88; *P*=.004); the figure for women was 1.71, (95% CI 1.11 to 2.64; *Z*=2.41; *P*=.016). | - Reduced alcohol consumption - Increased accessibility | TeleCoach app | Sweden | RCT time series 3 arms, I1=93, I2=93, C=144; no effect size reported |
| Barrio et al [25] | n=24; adults, (12 male 50%, 12 female 50%); mean age 48 years, attrition not reported | mHealth | - Mean comparisons - Paired *t* test and chi-square | Significant reduction in number of drinks and frequency of drinking (binge drinking days in the last 6 weeks declined from 25 (SD 18.6) to 5.8 (SD 8), *P*<.001; mean daily alcohol consumption in standard units declined from 6.5 (SD 4.3) to 1.9 (SD 1.8), *P*<.001. | - Reduced alcohol consumption - Increased patient satisfaction | Soporte Innovador al paciente con Dependencia del Alcohol | Spain | Pilot study; no effect size reported |
| Johansson et al [26] | n=3898; adults (1871 male 48%, 2026 female 52%); mean age 41.88 years, attrition 2854 | eHealth | - Paired *t* test, chi-square, Cohen *d* - 53% showed a clinically significant change to a lower level of alcohol use (χ^2^=254.403; *P*<.001) | 53% showed a clinically significant change to a lower level of alcohol use. The mean alcohol consumption fell (*t*=22.841; *P*<.001) and the proportion with low-risk consumption rose to 40 %. | - Reduced alcohol consumption - Increased QOL - Reduced depression | - Alcohol Use Disorders Identification Test (AUDIT)/Drug Use Disorders Identification Test - WHO Quality of Life-BREF, Readiness to Change Questionnaire - Timeline follow back | Sweden | Observational, 2 arms, I=2314, C=1584; no effect size reported |
| Deady et al [27] | n=104; adults (42 male 40.4%, 62 female 59.6%); mean age 21.74 years; race not reported; attrition not reported | eHealth | - Independent sample *t* tests - The treatment group demonstrated a significant reduction in drinks per week from baseline to posttreatment follow-up (RR=0.46; 95% CI 0.32 to 0.65; *P*<.001) representing a large effect (*d*=1.07). | Statistically significant reductions in alcohol use quantity (*d*=0.99) and frequency (*d*=0.76) and improvement in depression symptom severity (*d*=0.71) | - Reduced alcohol consumption - Reduced depression | - Depression Alcohol Project and Self-Help for Alcohol and other drug use Depression - Total Alcohol consumed in previous week reporting system. | Australia | RCT, 2 arms, I=60, C=44; large effect size |
| Campbell et al [28] | n=507; adults (315 male 62.1, 192 female 37.9%); mean age 34.9 years; attrition not reported | eHealth | Means, SD, frequencies, percentages, Bonferroni adjustment. Abstinence effect was more pronounced among patients with a positive urine drug and/or breath alcohol screen at the point of study entry (n=228; OR=2.18; 95% CI 1.30 to 3.68; *P*=.003) | Reduced dropout from treatment (Hazard Ratio=0.72, 95% CI 0.57 to 0.92, *P*=.010), increased abstinence (OR=1.62, 95% CI 1.12 to 2.35; *P*=.010). Quality-adjusted life years gained was similar between groups | Reduced alcohol consumption | Therapeutic education system augmented CBTs | The United States | RCT, 2 arms, I=255, C=252; no effect size reported |
| Stoner et al [29] | n=76; adults (50 male 65.8%, 26 female 34.2%); mean age 35.5 years; 27 white 35.5%, 37 Hispanic; 48.7% attrition intervention (20) and control (19) | mHealth | Independent sample *t* tests; drinks per drinking day decreased from baseline (mean=10.0, SD=5.7) through days 1 to 28 (mean=4.9, SD=1.8; *t*_62_=7.4; *P*<.001) and 29 to 56 (mean=4.1, SD=4.5; *t*_52_=6.6; *P*<.001) | Adequate adherence in both groups (no statistically significant difference), but intervention group sustained adherence longer than control (mean=19 days; 95% CI 0.0 to 44.0) than those in the control group (mean=3 days; 95% CI 0.0 to 8.1) during the first month of treatmen*t* (*P*=.04) | Reduced alcohol consumption | Adaptive, Goal-directed Adherence Tracking and Enhancement | The United States | RCT, 2 arms, I=37, C=39; medium effect size |
| Staton-Tindall et al [30] | n=127; adults in rural communities on parole (103 male 81%, 24 female 19%); mean age 30.5 years; 124 white 98%; attrition=23 | 2-way video | Binomial and logistic regression | No statistical significance between groups; however, 3+ motivational enhancement therapy (MET) sessions significantly reduced days drinking 72% (AOR: 0.28; 95% CI 0.08 to 0.97), fewer drinks per week, and fewer days experiencing alcohol problems | Reduced alcohol consumption | Project MET through video used AUDIT | The United States | Pilot study; no effect size reported |
| Chih [31] | n=142; adults (86 male 60.6%, 56 female 39.4%); mean age 38 years; 116 white 82%; attrition not reported | mHealth | *t* tests and chi-square tests | Active A-CHESS users engage in more online social activities, used more sessions, viewed more pages, and used A-CHESS longer. Lapsers are more likely to be female (*P*=.005) | Increased cognition | A-CHESS | The United States | Observational study, single arm; no effect size reported |
| Chih et al [32] | n=304; adults (186 male 61%, 118 female 39%); mean age 38 years; 58 white 38% attrition | mHealth | Bayesian Network Model; the area under receiver operating characteristic curve (AUC) of 0.829 in the 10-fold cross-validation and 0.912 in the external validation | Effective modeling predicted lapse within 1 week of report; AUC of 0.912 with 95% CI 0.862 to 0.972; *P*<.001 | Reduced alcohol consumption | A-CHESS | The United States | RCT, 2 arm, equal arms; no effect size reported |
| Gamito et al [33] | n=60; adults (50 male 84%, 10 female 16%); mean age 45.45 years; race not reported; attrition=4 | mHealth | Analysis of covariance; Frontal Assessment Battery was significant (*F*1_, 39_=4.308; η2=.099; *P*=.045) | No statistical significance between groups. Both groups experienced statistically significant increase in cognitive abilities, mental flexibility, psychomotor processing speed, and attention ability (*P*<.05) | Increased cognition | Mental games on apps | Portugal | RCT, 3 arms, I1=22, I2=23, C=19; medium effect size |
| Gamito et al [34] | n=54; adults (45 male 83.3%, 9 female 16.7%); mean age 45.37 years; race not reported; attrition=14 | mHealth | ANOVA, factorial design (*F*_1,52_=20.68; eta-square=0.41; *P*<.001) | Intervention group showed a statistically significant increase in frontal-lobe functioning (*P*=.01). | Increased cognition | SG-based cognitive stimulation plus treatment as usual | Portugal | RCT, 2 arms, I=33, C=35; medium effect size |
| Rose et al [35] | n=21; adults (12 male 57%, 9 female 43%); mean age 46.5 years; 20 white 95%; attrition not reported | Telephone | Paired *t* tests and McNemar tests. Mean scores on the ECBI improved from baseline (29.6, SD=18.8) to post-CBT (41.4, SD=12.6; *t*_31_=2.80; *P*<.01, Cohen *d*=0.63), and to post- Alcohol Therapeutic Interactive Voice Response (telephone-based; 42.9, SD=12.7; *t*_31_=3.39; *P*<.002, Cohen *d* <0.71). | Increased self-awareness, increased abstinence rate (*P*<.03), increased self-efficacy and coping (*P*<.01). | Reduced alcohol consumption | 12 weekly 90-min group CBT sessions | The United States | Pilot study, single arm; large effect size |
| McTavish et al [36] | n=349; adults (211 male 60.6%, 138 female 39.4); mean age 38.3 years; 289 white 82.9%; attrition=37 | mHealth | Paired *t* tests | Participants with alcohol and drug dependence showed lower levels of system use than those with only alcohol dependence (*t*_160_=2.139; *P*=.034) but had significantly more use of My Team service (*t*_157_=3.071; *P*=.003). Participants with mental health disorders showed lower levels of system use. | Increased cognition | A-CHESS | The United States | RCT, 2 arms, I=170, C=179; no effect size reported |
| Smit et al [37] | n=1,216,000; adults (987,000 male 81.14%, 267,000 female 18.86%); race and age not reported | eHealth | Frequencies, SDs, means comparisons | No statistical difference between treatment groups, but the new health care system offers much better returns on investment | Decreased cost | Alcohol model | The Netherlands | Secondary data analysis. Effect size ranged from small to medium across measures |
| McKay et al [38] | n=252; adults (162 male 64.3%, 90 female 35.7%); mean age 43.0 years; 224 black 88.9; attrition=10 | Telephone | Kruskal-Wallis tests and chi-square | No statistical significance between groups (χ^2^_2_=0.41; *P*=.81): TMC stronger significance for women | Reduced alcohol consumption | Diagnostic and Statistical Manual of Mental Disorders | The United States | RCT, 3 arms, I1=83, I2=83, C=86; no effect size reported |
